# The Kyoto Encyclopedia of Genes and Genomes—KEGG

**DOI:** 10.1002/(SICI)1097-0061(200004)17:1<48::AID-YEA2>3.0.CO;2-H

**Published:** 2000


**Yeast**
*Yeast* 2000; **17**: 48–55.

**Website Review**

### The Kyoto Encyclopedia of Genes and Genomes—KEGG

### http://www.genome.ad.jp/kegg/

**The KEGG project is run by the Institute for Chemical Research at Kyoto University, as part of the Japanese Human Genome Program. The page was constructed by a large project team (see** http://www.genome.ad.jp/kegg/kegg1.html**) and is maintained by Professor Minoru Kanehisa and Susumu Goto (Institute for Chemical Research). All screen views from the website are reproduced with the kind permission of Professor Minoru Kanehisa.**

### Page structure

The site (**http://www.genome.ad.jp/kegg/kegg2.html**) is organized into three sections: pathway information, genomic information, and computational tools. The pathway information section includes searchable pathway maps and orthologue tables of metabolic and regulatory pathways. There are also extensive catalogues of diseases (human), organisms (completely sequenced genomes or chromosomes), cells (cell lineages), enzymes and 'compounds'. All of these are extensively supported by searches of the relevant sections of the KEGG databases, which can be used to help with the correct specification of search terms.

The genomic information section is broken down into two parts, gene catalogues and Java map browsers. The 'gene catalogues' section is a comprehensive listing of all sequenced genes, for each organism, ordered by species. There are KEGG pages, which have all the genes present in the metabolic and regulatory pathway resources of KEGG, ordered by pathway. There are also links to pages that have the original functionally categorized lists of genes, as defined by the relevant sequencing consortia. The 'Java map browsers' section provides genome maps, and a tool that will draw a comparative map between two specified genomes. Currently, the expression map tools only allow users to query the expression data produced using the yeast microarrays. This section will no doubt be expanded as more microarray-based expression analysis is performed.

The computational tools section provides BLAST and FASTA searches of the gene and genome catalogues of KEGG.

### Page guide

### Pathway information

#### Pathway maps and orthologue tables

This section provides metabolic and regulatory pathway maps and tables of orthologous genes, the pathways can also be searched using the tools provided.

The metabolic pathway link leads to an ordered table of known metabolic pathways. Clicking on the pathway of interest leads to a flow-chart of the 'standard pathway' (Figure 1), in which all enzymes (Figure 2), substrates, products (Figure 3) and allied pathways are linked to pages on these topics. At the top of the page is an option box for changing to a view of the pathway in a chosen organism. This provides a picture of the pathway in which the enzymes whose genes (or putative functional homologues) have been sequenced in that organism are picked out in green. From the pathway page there is also a link to the orthologue table for that pathway, which shows any genes from that pathway that have been clustered to localized areas in the genomes of one or more organisms (Figure 4). Each cluster is denoted by a different background colour in the table. The table provides further links to organism-specific data, such as the locations of the genes in the genome, and the sequence files of the genes. The pathway page also offers a 'linkDB' search, which scans a whole host of databases for entries that relate to the pathway, pinpointing an enormous amount of information on the pathway of interest in a variety of species.

The regulatory pathway link leads to a categorized list of regulatory pathways. Links are provided to views of the pathway in various organisms and

Copyright © 2000 John Wiley & Sons, Ltd.

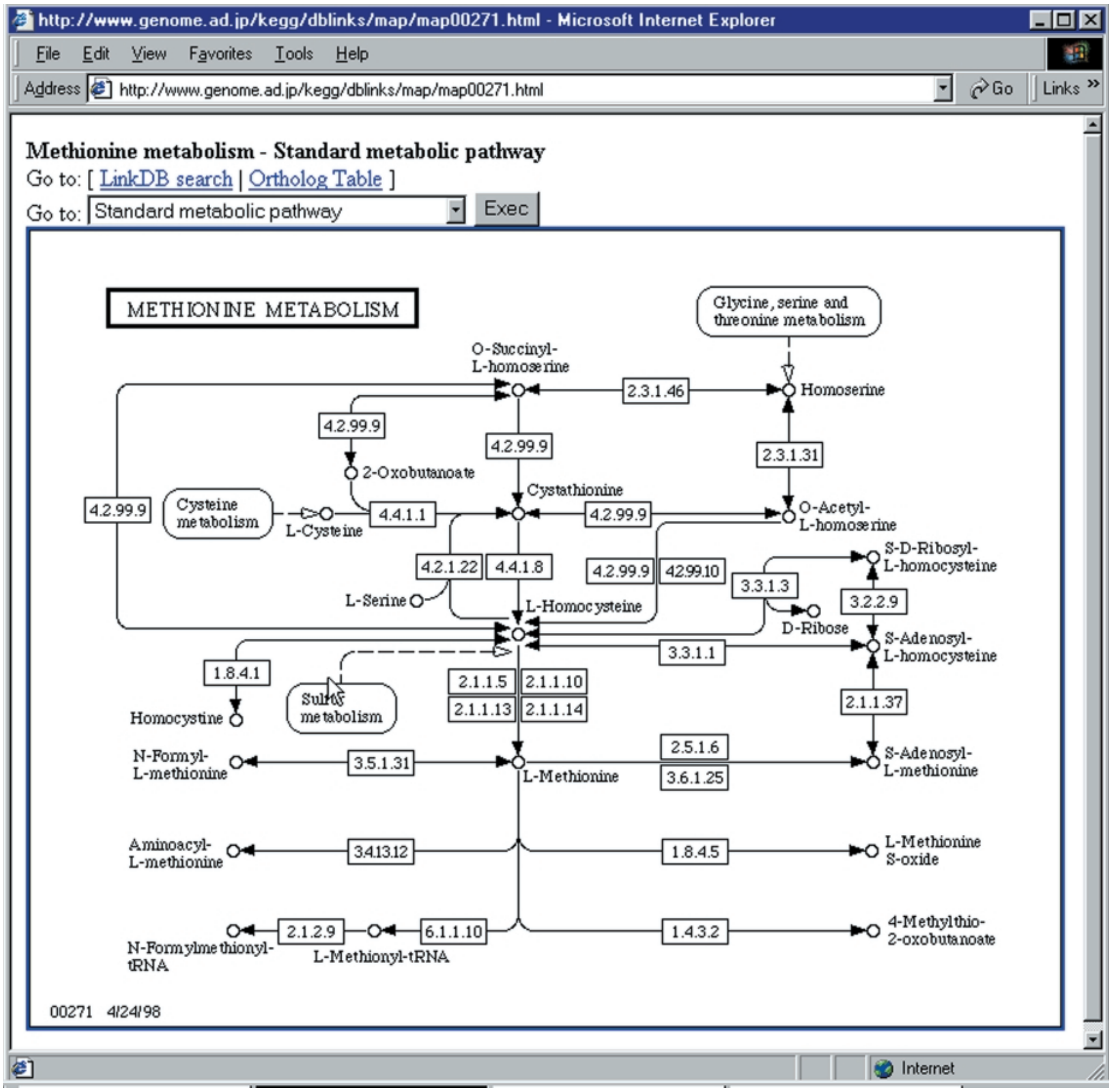

**Figure 1.** The KEGG 'standard pathway' for methionine metabolism. Each pathway page has a link to a 'linkDB' database search and the orthologue tables. The lower 'Go to' box allows users to nominate an organism; clicking the exec button produces a view of the pathway, with the genes mapped in that organism highlighted in green. Each enzyme, compound and pathway in the diagram is also linked to further information. (Reproduced with the kind permission of Professor Minoru Kanehisa). URL: **http://www.genome.ad.jp/kegg/dblinks/map/map00271.html**

also to the relevant orthologue table(s) directly from the list. Many of these pathways are illustrated as highly complex diagrams with multiple colours, depending upon the depth of knowledge about the components.

The tools available in this section include searches of the pathway maps and orthologue tables using gene names (supported by a search of the gene catalogue for correct gene names), including the option to highlight the gene(s) of interest in a chosen colour. It is possible to search either dataset, in a given organism, for genes with similarity to a given sequence and also to attempt to find a pathway linking two chosen compounds (supported by a search of the ligand catalogue for correct compound identifiers).

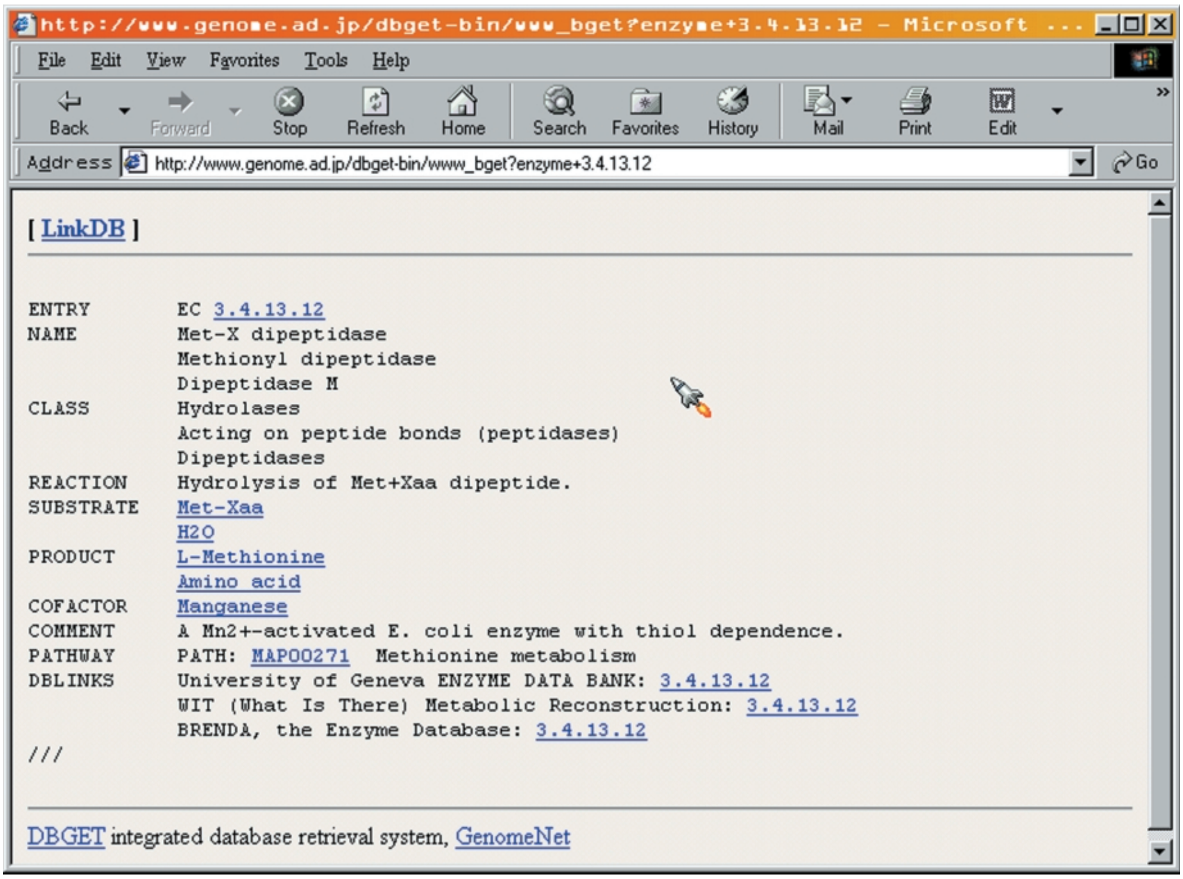

**Figure 2.** Detailed information on enzyme 3.4.13.12. Clicking on the box marked 3.4.13.12 in the methionine metabolism pathway leads to this page, which provides links to further information on this enzyme in several databases. (Reproduced with the kind permission of Professor Minoru Kanehisa). URL: **http://www.genome.ad.jp/dbget-bin/www_bget?enzyme + 3.4.13.12**

### Disease catalogues, cell catalogues and molecule catalogues

The disease section has the table of the International Classification of Diseases, the OMIM (Online Mendelian Inheritance in Man) tables of mapped human disease genes, ordered by chromosome, and the OMIM list of human diseases (with the genetic locations to which possible or proven susceptibility genes have been mapped). Although they are of course mainly of interest to those working on the human or higher mammal genomes, these tables are a valuable resource for geneticists.

The organisms section contains a list of all of the publicly available completely sequenced genomes (or whole chromosomes), ordered by organism, each of which is linked to the sequence file. There are also links to the entries for the original research articles in NCBI's PubMed Database and to the databases that originally hosted the sequence files. There is a separate viral genomes catalogue and the section also includes a taxonomic listing of all the other genome sequencing projects, with links to the sites of the consortia involved in each project. This well-organized and clearly presented section is of value to all those interested in comparative mapping and genomics.

The cell section provides access to the four currently available cell lineage maps. These are lists of every cell in the organism, as defined by its lineage, which can be expanded to achieve a description of each individual cell.

The enzyme section contains the EC number list and tables of enzymes as classified by their PIR

Copyright © 2000 John Wiley & Sons, Ltd.

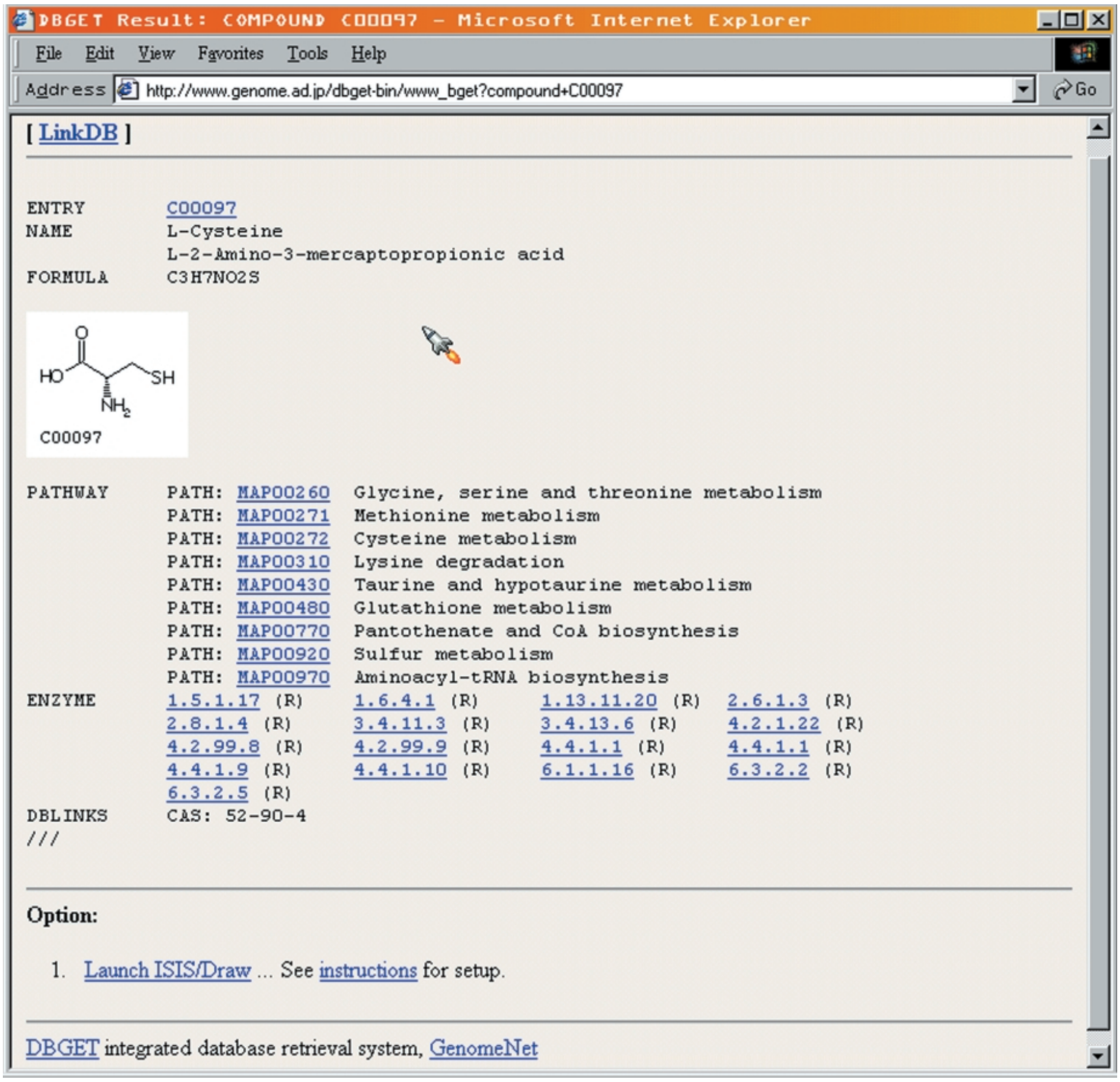

**Figure 3.** Further information on L-cysteine. Clicking on the small circle above L-cysteine in the methionine metabolism pathway leads to this page, which provides links to all the pathways in which it is involved and all the enzymes that produce or modify it. (Reproduced with the kind permission of Professor Minoru Kanehisa). URL: **http://www.genome.ad.jp/dbget-bin/www_bget?compound + C00097**

(protein information resource) superfamilies, the Prosite motifs they contain, or by their predicted or observed three-dimension folds (structural classification of proteins—SCOP).

The compounds section has a table giving the classification of all 'compounds with a biological role', with links to the pathways they occur in, the enzymes that utilize or modify them and also to the structures of the compounds, when they are known. There is also a copy of the periodic table available in this section.

### Genomic information

### Gene catalogues

This section contains lists of all sequenced genes for each organism, ordered by pathway or functional category. Keyword searches of the organism-

Copyright © 2000 John Wiley & Sons, Ltd.

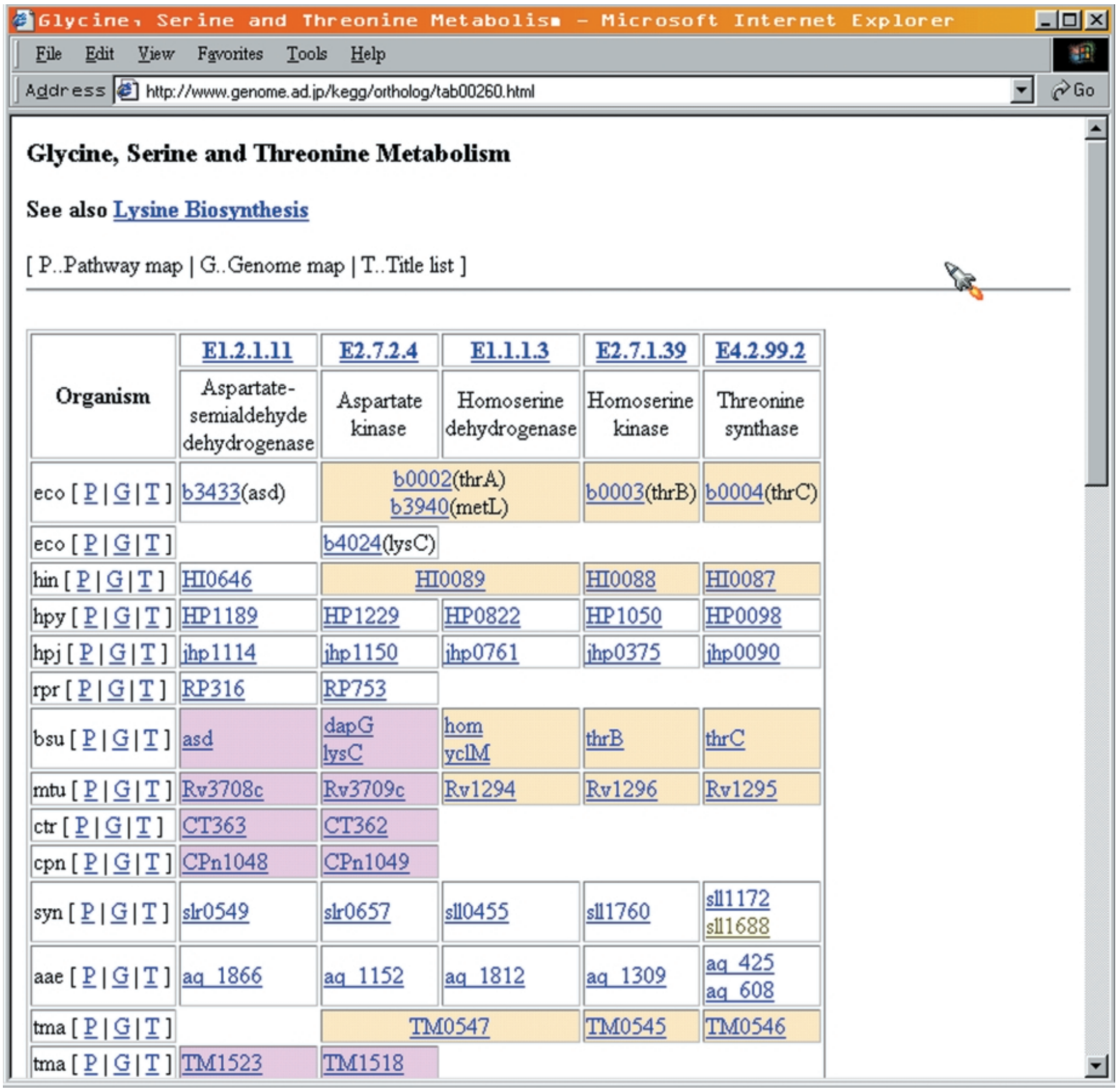

**Glycine, Serine and Threonine Metabolism**

**See also Lysine Biosynthesis**

[ P..Pathway map | G..Genome map | T..Title list ]

| Organism | E1.2.1.11 Aspartate-semialdehyde dehydrogenase | E2.7.2.4 Aspartate kinase | E1.1.1.3 Homoserine dehydrogenase | E2.7.1.39 Homoserine kinase | E4.2.99.2 Threonine synthase |
|---|---|---|---|---|---|
| eco [ P \| G \| T ] | b3433(asd) | b0002(thrA) b3940(metL) | | b0003(thrB) | b0004(thrC) |
| eco [ P \| G \| T ] | | b4024(lysC) | | | |
| hin [ P \| G \| T ] | HI0646 | HI0089 | | HI0088 | HI0087 |
| hpy [ P \| G \| T ] | HP1189 | HP1229 | HP0822 | HP1050 | HP0098 |
| hpj [ P \| G \| T ] | jhp1114 | jhp1150 | jhp0761 | jhp0375 | jhp0090 |
| rpr [ P \| G \| T ] | RP316 | RP753 | | | |
| bsu [ P \| G \| T ] | asd | dapG lysC | hom yclM | thrB | thrC |
| mtu [ P \| G \| T ] | Rv3708c | Rv3709c | Rv1294 | Rv1296 | Rv1295 |
| ctr [ P \| G \| T ] | CT363 | CT362 | | | |
| cpn [ P \| G \| T ] | CPn1048 | CPn1049 | | | |
| syn [ P \| G \| T ] | slr0549 | slr0657 | sll0455 | sll1760 | sll1172 sll1688 |
| aae [ P \| G \| T ] | aq_1866 | aq_1152 | aq_1812 | aq_1309 | aq_425 aq_608 |
| tma [ P \| G \| T ] | | TM0547 | | TM0545 | TM0546 |
| tma [ P \| G \| T ] | TM1523 | TM1518 | | | |

**Figure 4.** A section of the orthologue table for glycine, serine and threonine metabolism genes. The orthologues are listed by organism and groups of genes that are clustered in a genome are indicated by the coloured boxes. Each colour denotes a different cluster of genes. (Reproduced with the kind permission of Professor Minoru Kanehisa). URL: **http://www.genome.ad.jp/kegg/ortholog/tab00260.html**

specific subsections of the KEGG genes database are also provided.

Once the link to a KEGG page for a specific organism is chosen, a categorized list of the genes is provided. Clicking on a pathway expands the view to give a list of all the genes known from that pathway in that organism. Once a page with a functionally categorized gene list is chosen, a clickable list of categories is offered, which can be successively expanded to achieve a list of all the genes in a given pathway or functional grouping.

### Java map browsers

In the genome section, the genome maps link leads to a list of genome map browsers ordered by organism. The list of genomes includes several bacteria and archaea, *Saccharomyces cerevisiae* and the mouse. These provide a view of the entire

Copyright © 2000 John Wiley & Sons, Ltd. 

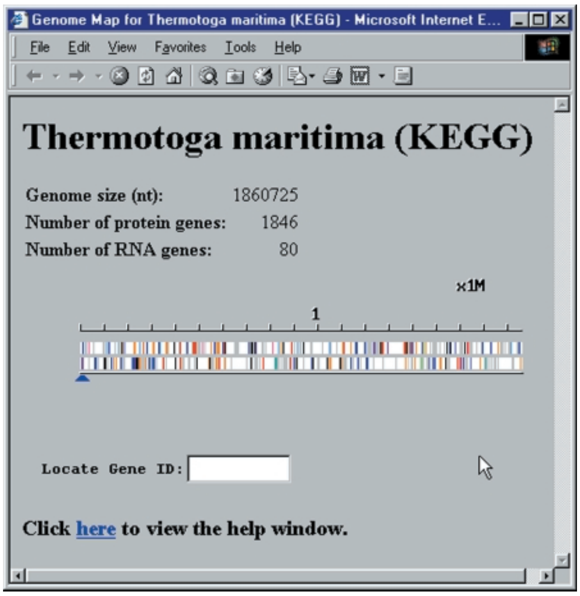

**Figure 5.** The KEGG genome map of *Thermotoga maritima*. The genome maps provide some basic details of the genome and a map where each line is a gene, the colour of the line indicating the functional category of the gene. A location search for any gene of choice is offered on the page. (Reproduced with the kind permission of Professor Minoru Kanehisa). To obtain a genome map, go to: **http://www.genome.ad.jp/kegg/java/launcher.html** and click on the button for the map you require

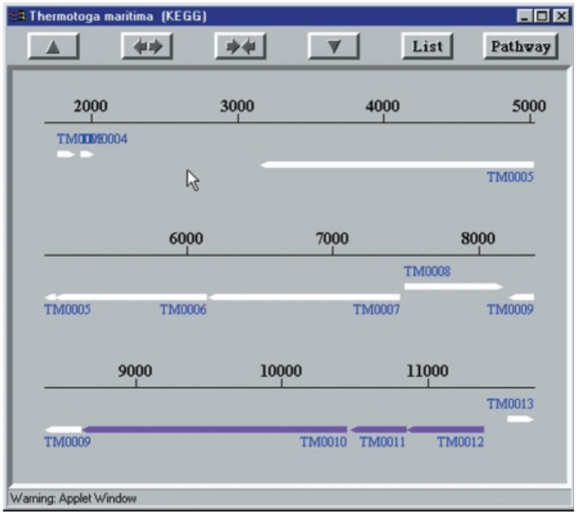

**Figure 6.** The Java applet window for the KEGG *Thermotoga maritima* genome map. This window provides a zoomed in view of the genome, which can be moved along the chromosome and zoomed in and out using the buttons at the top. The genes are coloured according to their functional class, in this case, the white genes are unclassified and the three clustered purple genes have roles in oxidative phosphorylation. There are also buttons which link to a list of all the genes in the window and the pathway(s) in which they are involved. (Reproduced with the kind permission of Professor Minoru Kanehisa). To obtain a genome map, go to: **http://www.genome.ad.jp/kegg/java/launcher.html** and click on the button for the map you require genome and a gene locator function (Figure 5). In a second Java applet window, a zoomed-in view is provided, with each gene colour-coded according to its functional category (Figure 6). Buttons at the top of the screen are used to move around the genome or to zoom in or out. There are also options to view a list of the genes in the region on display or to view the pathway in which they are involved.

There is also the option to draw a genome map comparison between two species, selected from a list, which consists of a selection of bacteria and archaea and *S. cerevisiae*, with a specified threshold for hits. This tool is best used in conjunction with the on-line manual, since the display produced is very complicated and lacks annotation. Again, a Java applet window shows a zoomed-in view of a small area, indicated by a blue box (which can be moved around) on the overall homology map.

Several other tools are offered, such as a search for the positions of chosen genes in a chosen genome. This produces a figure with arrows indicating the locations of the genes on the chromosomes. It can also give the sequence coordinates of each gene. Clicking on any of the gene indicators yields a second applet window with a zoomed-in gene map of the region around the chosen gene. It is also possible to specify the colours in which the chosen genes will be highlighted on the map.

Searches for gene clusters in two chosen genomes or in a user-selected subset of the completed genomes are also available in this section. These tools are an impressive resource. This section will be of great interest to anyone working on functional analysis or comparative mapping in microbes.

In the expression section, it is currently only possible to view the expression map generated for *S. cerevisiae* in the original Stanford experiments (DeRisi *et al.*, 1997). It is possible to choose the growth conditions from those analysed so far (and a time point, when available) and also to set a

Copyright © 2000 John Wiley & Sons, Ltd.

threshold level for the results to be displayed. The comparison tool can perform a search for genes whose expression levels at different time points during the diauxic shift or sporulation experiments vary by more than a chosen threshold value. There is also an option to cluster genes by their expression pattern during diauxic shift. This section will mainly be of interest to yeast researchers, until such time as data from other organisms is incorporated.

### Computational tools

In this section, BLAST and FASTA searches against the gene catalogue or the genome catalogue held in the KEGG database are available. In each case, these searches can be performed against the whole database or against selected organisms or species.

### Summary

KEGG is a highly structured and exceptionally comprehensive site, with something for everyone. In many ways it is perhaps best seen as a portal, since there are, of course, more specialized sites for particular organisms. As with other such sites, many of the gene annotations and designations are based on similarity search alone, rather than experimental measurements. It is known that as many as 8% of these designations may be incorrect (Brenner, 1999), and the problems may become worse as we begin to acquire more genomic data from higher organisms (Wheelan et al., 1999). Indeed, a major problem is that the functional classes themselves are often inhomogeneous and inadequately defined (Kell and King, 2000). Users should consequently look to cross-check the analyses provided. KEGG is arguably best when working with the central pathways of metabolism, since some of the more arcane areas, such as terpenoid metabolism (**http://www.genome.ad.jp/kegg/dblinks/map/map00900.html**), lack the very useful orthologue information. Another drawback of the pathway system is that it is designed merely to show which genes from a pathway have been sequenced in each organism and does not indicate when a pathway is absent in an organism, e.g. if you go to the sterol pathway and ask for the pathway in *E. coli*, you get the pathway map with several enzymes coloured in green, even though *E. coli* does not make sterols (**http://www.genome.ad.jp/dbget-bin/get_pathway?org_name=eco&mapno=00100**).

In general, the Java applets downloaded very quickly (the slowest taking about 30 seconds) and were easy to manipulate. However, several contained complex displays without legends, in particular the genome comparison figures. These are best interpreted by using the help button, to link to the on-line help manual, before viewing the display.

### Equipment details

This review was completed using a Dell PIII 500MHz PC with a Pentium processor, running Windows NT version 4.0, with a permanent 10 Mbps Ethernet and Internet link and a screen with 1024 × 768 pixels resolution. The primary software used was Internet Explorer version 5; however, several of the more complex pages have also been accessed using Netscape Communicator version 4.7. 10 Mbps Ethernet links are fast, but are fairly common in academic institutions, so any differences in the speed of downloading applets, etc. will most likely be due to high usage of the connection to the Internet (this review was completed out of term-time). The resolution of the screen, however, is higher than average and so readers may find that they will need to scroll around a significant amount to see the entirety of the larger metabolic and regulatory pathway figures.

### KEGG source websites

OMIM: **http://www.ncbi.nlm.nih.gov/Omim/**
SCOP: **http://scop.mrc-lmb.cam.ac.uk/scop**
PROSITE: **http://expasy.hcuge.ch/sprot/prosite.html**
PIR: **http://pir.georgetown.edu/** (mirror at MIPS)
IUPAC/IUBMB: **http://www.chem.qmw.ac.uk/iupac/jcbn/**
NCBI taxonomy list: **http://www.ncbi.nlm.nih.gov/Taxonomy/**
GDB: **http://gdbwww.gdb.org/**
MGD: **http://www.informatics.jax.org/**
Flybase: **http://flybase.bio.indiana.edu/**
MIPS: **http://www.mips.biochem.mpg.de/proj/yeast/**
SGD: **http://genome-www.stanford.edu/Saccharomyces/**
Pasteur Institute genomes: **http://bioweb.pasteur.fr/docs/gendocdb/banques.html**
All TIGR genomes: **http://www.tigr.org/**

Copyright © 2000 John Wiley & Sons, Ltd.

*Rikettsia* genome: **http://evolution.bmc.uu.se/~siv/gnomics/Rickettsia.html**
*M. pneumoniae* genome: **http://www.zmbh.uni-heidelberg.de/M_pneumoniae/MP_Home.html**
All Sanger Centre genome projects: **http://www.sanger.ac.uk/Projects/**
*Chlamidiae* genomes at Berkeley: **http://chlamydia-www.berkeley.edu:4231/**
*Synechocystis* genome: **http://www.kazusa.or.jp/cyano/cyano.html**
*M. thermoautotrophicum* genome: **http://www.cric.com/genesequences/methanobacter/abstract.html**
*P. horikoshii* genome: **http://www.bio.nite.go.jp/ot3db_index.html**
*P. abyssi* genome: **http://www.genoscope.cns.fr/Pab/**

### Metabolomics-related websites

ExPASy—a very useful on-line version of the classic metabolic pathway maps: **http://www.expasy.ch/cgi-bin/search-biochem-index**

EcoCyc—an encyclopedia of *E. coli* genes and metabolism: **http://ecocyc.pangeasystems.com/ecocyc/ecocyc.html**

GeneCards—a database of human genes, proteins and diseases: **http://bioinformatics.weizmann.ac.il/cards/**

The E-cell project—integrating genomics and metabolism: **http://e-cell.org/**

Main metabolic pathways on Internet—also has a searchable, downloadable version: **http://home.wxs.nl/~pvsanten/mmp/mmp.html**

Minnesota Biocatalysis/Biodegradation Database—primarily pathways for xenobiotic, chemical compounds: **http://www.labmed.umn.edu/umbbd/index.html**

PathDB—a metabolic pathway database: **http://www.ncgr.org/research/pathdb/** which will link with Gepasi: **http://gepasi.dbs.aber.ac.uk/softw/gepasi.html** a metabolic pathway and biochemical kinetics simulator written by Pedro Mendes in Aberystwyth and now being further developed at the NCGR: **http://www.ncgr.org/software/gepasi/index.html**

WIT—a server linking genes and metabolism: **http://wit.mcs.anl.gov/WIT2/**. This includes metabolic pathways based on the Enzymes and Metabolic Pathways database: **http://www.empproject.com/**

DBSolve—software for metabolic, enzymatic and receptor–ligand binding simulation: **http://websites.ntl.com/~igor.goryanin/**

Source: Metabolomics in Aberystwyth: **http://gepasi.dbs.aber.ac.uk/dbk/metabol.html**—this page also has lists of links to genomics, proteomics, transcriptomics and metabolomics pages and also to functional catalogues. Author: Douglas Kell.

### References

Brenner SE. 1999. Errors in genome annotation. *Trends Genet* **15**: 132–133.

DeRisi JL, Iyer VR, Brown PO. 1997. Exploring the metabolic and genetic control of gene expression on a genomic scale. *Science* **278**: 680–686.

Kell DB, King RD. 2000. On the optimization of classes for the assignment of unidentified reading frames in functional genomics programmes: the need for machine learning. *Trends Biotechnol* **18**: 93–98.

Wheelan SJ, Boguski MS, Duret L, Makalowski W. 1999. Human and nematode orthologs—lessons from the analysis of 1800 human genes and the proteome of *Caenorhabditis elegans*. *Gene* **238**: 163–170.

Some of the sites reviewed will already be known to you, but perhaps their content will be less well known. The Website Review is intended to help you discover new sites of interest, but also to provide a rapid and convenient means of revealing what you always knew was there but never had the time or inclination to look at. These articles are a personal critical analysis of the website. If you have any information about sites you think are worthy of being more widely known, the Managing Editor would be pleased to hear from you.

This review was written by Dr Joanne Wixon (Managing Editor) and Professor Douglas Kell (Section Editor, Metabolomics) **http://gepasi.dbs.aber.ac.uk/dbk/metabol.html**

Copyright © 2000 John Wiley & Sons, Ltd. 
